# Retraction Note: Observational study: daily treatment with a new compound “tradamixina” plus serenoa repens for two months improved the lower urinary tract symptoms

**DOI:** 10.1186/s12893-016-0139-0

**Published:** 2016-04-28

**Authors:** Fabrizio Iacono, Domenico Prezioso, Ester Illiano, Antonio Ruffo, Giuseppe Romeo, Bruno Amato

**Affiliations:** Department of Urology, University Federico II of Naples, Via S. Pansini, 5, Naples, 80131 Italy; Department of General, Geriatric, Oncologic Surgery and Advanced Technologies, University “Federico II” of Naples, Via Pansini, 5, Naples, 80131 Italy

## Retraction Notice

This article [1] has been retracted by the authors because it contains large portions of text that were duplicated from a number of pervious publications including Chapple et al. [2], Untergasser et al. [3] and Mariotto et al. [4]. The authors apologise for failing to cite these articles.

## Notes

The online version of the original article can be found at 10.1186/1471-2482-12-S1-S22.
